# Diet-Induced Amyloid Precursor Protein Dysregulation in Kidney and Adipose Tissue Mediates Mitochondrial Dysfunction

**DOI:** 10.3390/cells15111033

**Published:** 2026-06-04

**Authors:** Alexandré Delport, Ebrahim Ally, Shantal Maharaj, Raymond Hewer

**Affiliations:** 1 Discipline of Biochemistry, School of Agriculture and Science, University of KwaZulu-Natal, Pietermaritzburg 3201, South Africahewerr@ukzn.ac.za (R.H.); 2 Animal House, School of Agriculture and Science, University of KwaZulu-Natal, Pietermaritzburg 3201, South Africa

**Keywords:** amyloid precursor protein, βC-terminal fragment, high fat diet, kidney tissue, adipose tissue

## Abstract

While amyloid precursor protein (APP) overexpression in adipose tissue is a recognized consequence of high-fat diet (HFD) feeding, its role in metabolically active organs and the mechanisms linking it to systematic dysfunction remain unclear. In particular, the potential for diet-induced APP dysregulation in the other tissues and the contribution of its βC-terminal fragment (βCTF) are poorly characterized. Using a high-fat diet (HFD) mouse model to induce systematic metabolic stress, we assessed APP and βCTF levels across multiple tissues. HFD triggered a tissue-specific response, with APP levels increasing >2-fold in visceral and subcutaneous white adipose tissue (WAT) and in the kidney but remained unchanged in the liver and brain. βCTF levels were significantly elevated in the visceral WAT (3-fold) and kidney. In these responsive tissues, APP and βCTF accumulated within mitochondria, which coincided with significantly reduced complex I and IV activities. Complementary in vitro studies confirmed that APP levels can dictate mitochondrial function. Furthermore, we identified that cytokines–IL-4, IL-13, TNF-α, and IL-1β–induced *APP* transcription, providing a mechanistic link between diet-induced inflammation and APP dysregulation. Collectively, our findings demonstrate that APP is overexpressed in response to HFD in select peripheral tissues, which coincides with reduced mitochondrial complex enzyme activities and increased cytokine levels.

## 1. Introduction

The amyloid precursor protein (APP) is most widely recognized for its central role in the pathogenesis of Alzheimer’s disease (AD); however, it has also been implicated in several other pathological conditions. In these instances, overexpression of the protein has been reported. For instance, elevated APP levels have been documented in brain tissue following traumatic brain injury [1], in the muscle tissue of patients with amyotrophic lateral sclerosis [2], in liver tissue after chronic alcohol intake [3], and, notably, in the adipose tissue of obese and diabetic individuals [4,5].

The connection is particularly pronounced in the context of metabolic stress. Multiple studies have demonstrated that APP is overexpressed in white adipose tissue (WAT) in rodent models of diet-induced obesity (DIO) following high-fat diet (HFD) exposure, with its levels correlating with weight gain [5,6,7]. Importantly, this overexpression has direct functional consequences. An and colleagues [5] demonstrated that overexpressed APP in WAT mislocalizes to the mitochondria, where it impairs protein import and reduces mitochondrial respiration [8,9]. This finding aligns with other reports linking APP upregulation to mitochondrial dysfunction, oxidative stress, and apoptosis [10,11]. Furthermore, the βC-terminal fragment (βCTF) of APP, a proteolytic product generated during amyloidogenic processing, is itself a key contributor to mitochondrial impairment [12]. In AD models, βCTF accumulates in the mitochondria and disrupts their function [10,11]. Despite its established toxicity in neuronal contexts, the accumulation and potential role of βCTF in peripheral metabolic tissues during obesity remains largely unexplored.

Prolonged HFD intake induces systemic metabolic stress, damaging multiple organs beyond adipose tissue. Lipid accumulation can lead to hepatic steatosis and lipotoxicity in the liver, as well as mitochondrial dysfunction associated with renal impairment in the kidneys [13,14,15,16]. HFD also triggers neuroinflammation and disrupts synaptic plasticity and glucose homeostasis in the brain [17]. However, unlike the consistent upregulation observed in WAT, increases in APP levels or its mRNA have not been reliably reported in the liver, brain, or kidney tissue following HFD [5,7]. This suggests that APP overexpression may be a tissue-specific response to metabolic stress, the full scope of which is not yet understood.

Given the systemic effects of HFD and the established association between APP and cellular dysfunction, key questions remain. Is APP upregulation a broader phenomenon in DIO, occurring in other metabolically stressed tissues such as the kidney? Does this overexpression drive the production of the toxic βCTF fragment in peripheral tissues, and could this contribute to the mitochondrial dysfunction observed in obesity? Furthermore, what are the upstream mediators of APP overexpression in this context? Therefore, this study aimed to systematically investigate APP and βCTF levels across multiple tissues in a DIO model; determine the subcellular localization of APP/βCTF and its functional impact on mitochondria; and assess whether cytokines upregulated by HFD can directly regulate *APP* transcription.

## 2. Materials and Methods

### 2.1. Animal Housing and Husbandry

All animal procedures were approved by the Animal Research Ethics Committee of the University of KwaZulu-Natal (AREC/00003469/2023) and by the national authority (DALLRD) and were performed in accordance with institutional and national guidelines by a South African Veterinary Council-accredited individual (AL18/16976). Male C57BL/6 mice were obtained post-weaning (3 weeks old) from the Africa Health Research Institute (AHRI, South Africa) and housed under a 12 h light/dark cycle at 25 ± 2 °C. A sample size of *n* = 4 per group was determined by power calculation incorporating a 10% attrition rate and the 3Rs principle. All included data form the complete cohort.

### 2.2. Animal Diets and Weight Analyses

Upon arrival, mice were weighed and randomly assigned to two groups (*n* = 4 per group): normal diet (ND) or high-fat diet (HFD). Mice were housed in pairs in standard EU Type 3 cages with ad libitum access to water. The ND group received a standard rodent diet (20.0% protein, 8.0% fat; Avi-Plus Rodent Cubes, Avi Products, Cato Ridge, South Africa). The HFD group received the standard chow supplemented with continuous access to a high-fat supplement paste (64.5% fat; see Table S1 for full composition). This dietary intervention was maintained for 25 weeks to establish obesity-associated metabolic phenotypes [16]. Body weight was measured weekly, and weight gain was calculated from baseline (day 0). Food intake of the HFD supplement was monitored. To minimize stress-related variability, the order of weighing and cage placement was maintained consistently throughout the study.

### 2.3. Intraperitoneal Glucose Tolerance Test

An intraperitoneal glucose tolerance test (IPGTT) was performed at the end of the dietary intervention. Following a 12 h fast, mice received an intraperitoneal (IP) bolus of glucose (1 g/kg body weight). Prior to blood collection, the tail vein was treated with a topical anesthetic (5 mM lidocaine, MilliporeSigma, Burlington, MA, USA, L7757) and warmed for one minute. Blood was collected via tail vein micro-sampling at 0, 15, 30, 60, 90, and 120 min post-injection, and glucose concentration was measured using a glucometer (Bionime Blood Glucose Meter GM550, Bionime Corporation, Taichung City, Taiwan). The order of glucose readings was kept consistent for all animals. The area under the curve (AUC) for each IPGTT was calculated using GraphPad Prism 11 (GraphPad Software Inc., San Diego, CA, USA).

### 2.4. Euthanasia and Tissue Harvesting

Mice were euthanized by cervical dislocation. Whole blood was subsequently collected via cardiac puncture from the thoracic cavity using a 1 mL syringe and a 27-gauge needle. Blood samples were allowed to coagulate for 30–60 min at room temperature (RT) and then centrifuged at 1500× *g* for 10 min at 4 °C. The serum supernatant was collected, aliquoted, and stored at −80 °C. Visceral WAT (vWAT), subcutaneous WAT (sqWAT), kidneys, liver, and brain were harvested, weighed, and flash frozen in liquid nitrogen.

### 2.5. Metabolic Tests

Serum levels of creatine and activities of creatine kinase (CK) and aspartate aminotransferase (AST) were quantified using commercial assay kits (MAK116, MAK055, and MAK080; MilliporeSigma, Burlington, MA, USA) following the manufacturer’s protocols.

### 2.6. Tissue Homogenization

Tissue homogenization was performed as previously described by An, Scherer [18], and Cote et al. [19], with modifications. Briefly, tissues were weighed (approximately 100 mg; 200 mg for vWAT) and minced in 700 µL lysis buffer (50 mM HEPES, 150 mM NaCl, 5 mM EDTA, 1% (*v*/*v*) Triton X-100, and pH 7.5) containing 1× protease inhibitor cocktail (PIC; Halt Protease Inhibitor Cocktail, EDTA-free, Thermo Fisher Scientific, Waltham, MA, USA). Perirenal adipose tissue was removed from kidneys prior to processing. Homogenization was carried out using a Dounce homogenizer (Thermo Fisher Scientific, Waltham, MA, USA) with 20 strokes, followed by incubation for 30 min on an end-over-end rotator at 4 °C. Samples were centrifuged at 18,000× *g* for 20 min at 4 °C. For kidney, liver, and brain tissues, the supernatant was collected as the whole-tissue lysate. For WAT, the lipid layer was removed, and the supernatant was transferred to a new tube. This centrifugation and transfer process was repeated until no visible lipid layer remained.

### 2.7. Tissue Subcellular Fractionation

Mitochondrial and post-mitochondrial fractions were isolated as previously described by Osto et al. [20], with modifications. Kidney (100 mg), sqWAT (200 mg), and vWAT (400 mg) tissues were minced in 700 µL hypotonic isolation buffer (20 mM HEPES, 70 mM sucrose, 220 mM mannitol, 5 mM KH_2_PO_4_, 5 mM MgCl_2_, 1 mM EDTA, and pH 7.4) containing 1× PIC. Tissues were homogenized with a Dounce homogenizer (20–25 strokes) and incubated on ice for 10 min. Homogenates were centrifuged at 1000× *g* for 5 min at 4 °C, and the supernatant was collected. This low-speed centrifugation was repeated twice to remove nuclei and intact cells. The supernatant was then centrifuged at 10,000× *g* for 10 min at 4 °C to pellet the mitochondrial fraction. The resulting supernatant was retained as the post-mitochondrial fraction. The mitochondrial pellet was washed twice by resuspension in isolation buffer and centrifugation at 10,000× *g* for 5 min at 4 °C. The final mitochondrial pellet was resuspended in isolation buffer containing 0.01% (*v*/*v*) Triton X-100. The post-mitochondrial fraction was further cleared by centrifugation at 12,000× *g* for 10 min at 4 °C (repeated twice) to remove residual mitochondrial contaminants.

### 2.8. Mammalian Cell Culture

HEK293 (ATCC^®^ CRL-1573) and 3T3-L1 (ATCC^®^ CL-173) cell lines (Cellonex, Johannesburg, South Africa) were maintained at 37 °C with 5% CO_2_ in DMEM supplemented with 10% (*v*/*v*) fetal bovine serum (FBS), 0.1× penicillin–streptomycin, and 50 μg/mL gentamicin (complete medium). The HEK-APP overexpressing cell line, generated previously [21], was cultured in complete medium containing 900 µg/mL G418 (MilliporeSigma, Burlington, MA, USA). For APP chemical knock down studies, the HEK293 cell line was treated with 0 (DMSO only), 10, 25, or 50 µM phenserine (MilliporeSigma, Burlington, MA, USA; P0111) for 24 h.

### 2.9. Whole-Cell Lysates and Subcellular Fractionation with Mammalian Cell Lines

HEK293 and HEK-APP overexpressing cell lines were grown in T75 flasks until >70% confluency. Cells were harvested and washed three times with phosphate-buffered saline (PBS; Thermo Fisher Scientific, Waltham, MA, USA; 18912014). For whole-cell lysates, 20% of the cell pellet was resuspended in Pierce^®^ RIPA buffer (Thermo Fisher Scientific, Waltham, MA, USA; 89900) containing 1× PIC and 1 U/mL DNase I (MilliporeSigma, Burlington, MA, USA; D4263) and incubated for 1 h at 4 °C with agitation. Samples were centrifuged at 18,000× *g* for 10 min and the supernatant was collected. For subcellular fractionation, the remaining 80% of the cell pellet was used. Mitochondrial and post-mitochondrial fractions were isolated using the Mitochondria Isolation Kit for Cultured Cells (Thermo Fisher Scientific, Waltham, MA, USA; 89874) according to the manufacturer’s instructions.

### 2.10. Western Blot

Protein concentration of mouse tissue samples was determined using the Bradford protein assay (MilliporeSigma, Burlington, MA, USA; B6916), while cell lysate concentration was determined using the Pierce^®^ BCA Protein Assay Kit (Thermo Fisher Scientific, Waltham, MA, USA; 23227), both according to the manufacturers’ instructions. Samples were separated by reducing SDS-PAGE alongside a Spectra™ Multicolor Broad Range Protein Ladder (Thermo Fisher Scientific, Waltham, MA, USA) and transferred to nitrocellulose membranes. Membranes were horizontally cut to probe for different proteins, ensuring at least two molecular weight markers were retained per section.

Membrane sections were probed with the following primary antibodies: rabbit anti-APP (tissue lysates, 1:2500; Cell Signaling Technology, Danvers, MA, USA; 76600, RRID: AB_2925222) or mouse anti-APP_C99 (cell lysates, 1:2500; MilliporeSigma, Burlington, MA, USA; MABN380, RRID: AB_2714163). Subsequently, membranes were incubated with species-matched secondary antibodies conjugated to horseradish peroxidase (HRP): goat anti-rabbit IgG (1:10,000; Jackson ImmunoResearch Laboratories, West Grove, PA, USA; 111-035-144, RRID: AB_2307391) or donkey anti-mouse IgG (1:10,000; Jackson ImmunoResearch Laboratories, West Grove, PA, USA; 715-035-150, RRID: AB_2340770). β-Actin was used as a loading control and was detected using an HRP-conjugated anti-β-actin antibody (1:15,000; MilliporeSigma, Burlington, MA, USA; A3854, RRID: AB_262011). For subcellular fractions, membranes were probed with a multiplex antibody cocktail: rabbit anti-AIF (D39D2) (1:2000; Cell Signaling Technology, Danvers, MA, USA; 5318, RRID: AB_10634755) and rabbit anti-MEK1/2 (D1A5) (1:1000; Cell Signaling Technology, Danvers, MA, USA; 8727, RRID: AB_10544537), followed by HRP-conjugated goat anti-rabbit IgG (1:10,000) [22].

Proteins were visualized using ECL reagent (Clarity Western ECL Substrate, Bio-Rad, Hercules, CA, USA) with equipment and settings as previously described [23]. Total protein staining was performed with amido black [24]. All image analyses were conducted with ImageJ software 1.53t [25]. Band intensities for whole-tissue lysates were normalized to β-actin. For subcellular fractionation, whole-cell lysate band intensities were normalized to the sum of AIF and MEK1/2 signals, all mitochondrial fractions to AIF, and all post-mitochondrial fractions to MEK1/2. Relative band intensity was expressed as fold change relative to the control sample, set to 1.

### 2.11. Mitochondria Enzyme Complex Activity Assays

The activities of mitochondrial enzyme complexes I, IV, and citrate synthase were measured as described by Spinazzi et al. [26]. Complex IV and citrate synthase activities were assayed in whole-tissue lysates from vWAT, sqWAT, kidney, liver, and brain of ND- and HFD-fed mice, as well as in whole-cell lysates from HEK293 and HEK-APP cell lines. Complex I activity was measured in mitochondria isolated from vWAT, sqWAT, and kidney of ND- and HFD-fed mice. Briefly, complex IV activity was assessed with 1 mM reduced cytochrome c, with and without 10 mM KCN, using 10 µg of tissue lysate or 50 µg of cell lysate by reading absorbance at 550 nm for 2 min. KCN inhibited the reaction in all cases. Citrate synthase activity was measured using 1 mM 5,5′-Dithiobis(2-nitrobenzoic acid) and 10 mM Acetyl CoA with 10 µg of tissue lysate or 50 µg of cell lysate by recording the absorbance at 412 nm for 2 min. Complex I activity was measured using 10 mM NADH and 10 mM ubiquinone, with and without 1 mM rotenone, reacted with 25 µg of isolated mitochondria by measuring absorbance at 340 nm for 2 min. Complex I activity was taken as rotenone-sensitive activity by subtracting rotenone-resistant activity from total activity. To express complex I and IV activities relative to mitochondrial abundance, the activity for each sample was normalized to the corresponding citrate synthase activity. To determine the decrease in complex I/CS or IV/CS ratio for vWAT, sqWAT and kidney tissue after HFD exposure, the I/CS or IV/CS ratio was subtracted from the mean I/CS or IV/CS for each tissue after ND exposure.

### 2.12. Mitochondrial Membrane Potential Assay

Mitochondrial membrane potential was measured using the cationic dye tetramethyl rhodamine ethyl ester perchlorate (TMRE; MilliporeSigma, Burlington, MA, USA; 87917) as previously described by Kalbáčová et al. [27], with modifications. Briefly, HEK293 and HEK-APP cell lines were seeded at 1 × 10^5^ cells/mL into poly-L-lysine-pretreated 96-well black-walled plates and grown for 24 h. Cells were washed twice with phenol red-free DMEM and then treated with either 10 µM carbonyl cyanide 4-(trifluoromethoxy) phenylhydrazone (FCCP; MilliporeSigma, Burlington, MA, USA; C2920) or an equivalent volume of DMSO for 10 min. Following treatment, 100 nM TMRE was added for 20 min. Cells were washed twice with phenol red-free DMEM, and 200 µL of phenol red-free DMEM was added to each well. Plates were equilibrated at RT for 10 min before fluorescence measurement using a GloMax^®^ Discover Microplate Reader (Promega, Fitchburg, WI, USA) with excitation at 520 nm and emission at 580–640 nm. The relative TMRE signal was calculated as a percentage of the signal from HEK293 cells treated with DMSO vehicle control.

### 2.13. Confocal Microscopy

HEK293 and HEK-APP cell lines were seeded at 0.5 × 10^5^ cells/mL in 12-well plates containing 18 mm poly-L-lysine-coated glass coverslips and grown for 24 h. Cells were fixed with 3.7% paraformaldehyde for 15 min at 37 °C, washed three times with PBS and permeabilized with 0.1% (*v*/*v*) Triton X-100 in PBS (PBS-T) for 15 min at RT. Coverslips were blocked with 1% BSA in PBS containing 0.05% (*v*/*v*) Tween-20 (PBS-T) for 1 h at RT, followed by incubation with primary antibodies overnight at 4 °C with gentle rocking: mouse anti-APP_C99 (1:1000; MilliporeSigma, Burlington, MA, USA; MABN380, RRID: AB_2714163) together with either rabbit anti-AIF (D39D2) (1:1000; Cell Signaling Technology, Danvers, MA, USA; 5318, RRID: AB_10634755) or rabbit anti-TOMM40 (1:1000; Thermo Fisher Scientific, Waltham, MA, USA; PA5110507, RRID: AB_2855918).

Coverslips were washed three times for 5 min with PBS-T and incubated with secondary antibodies for 1 h at RT: goat anti-mouse IgG conjugated to Alexa Fluor 488 (1:2000; Thermo Fisher Scientific, Waltham, MA, USA; A110011, RRID: AB_2534069) and donkey anti-rabbit IgG conjugated to Alexa Fluor 594 (1:2000; Thermo Fisher Scientific, Waltham, MA, USA; A-21207, RRID: AB_141637). After three additional 5 min washes with PBS-T, coverslips were mounted and stored at −20 °C until imaging.

All images were acquired using a Zeiss 710 Laser Scanning Microscope at the Microscopy and Microanalysis Unit, University of KwaZulu-Natal with Zen 2010 Software (version 6.0.0.320). Alexa Fluor 488 was excited at 488 nm with emission recorded at 493–595 nm, while Alexa Fluor 594 was excited at 561 nm with emission collected at 586–670 nm. Images were analyzed using ImageJ with the JACoP plugin v2.1.4 [28]. Manders’ and Costes’ coefficients were calculated to assess colocalization [29,30].

### 2.14. Cytokine Array

Whole-tissue lysates of vWAT and kidney tissue from ND- and HFD-fed mice were analyzed using a mouse cytokine array (RayBiotech, Peachtree Corners, GA, USA; AAM-TH17-1-8) according to the manufacturer’s instructions. Arrays were loaded with equal total protein concentrations (0.5 mg/mL). Signal intensity was quantified by densitometry using ImageJ software after 50-pixel background subtraction. The mean negative control value was subtracted from all detected spots, and values were normalized to the mean positive control intensity. Cytokine fold change was calculated by setting the signal from the first ND control sample to 1. Positive control signals (P1–P3) were consistent across arrays after normalization, confirming comparable array analysis.

### 2.15. qPCR

3T3-L1 and HEK293 cell lines were seeded at 0.75 × 10^5^ cells/mL in 96-well plates and grown for 24 h. Cells were treated with 5 ng/mL of the following cytokines for 24 h: mouse or human IL4 (MilliporeSigma, Burlington, MA, USA; I1020 or GF337), human IL13 (Thermo Fisher Scientific, Waltham, MA, USA; AF-200-13), TGF-β (Thermo Fisher Scientific, Waltham, MA, USA; 100-21), TNFα (Thermo Fisher Scientific, Waltham, MA, USA; 300-01A), or IL-1β (Thermo Fisher Scientific, Waltham, MA, USA; AF-200-01B). Following treatment, qPCR was performed using the Cells-to-Ct kit (Thermo Fisher Scientific, Waltham, MA, USA; A35377) according to the manufacturer’s instructions.

The following TaqMan assays were used: β-actin (Hs03023880_g1), mouse APP (Mm01344172_m1), and human APP (Hs00169098_m1). Reverse transcription reactions were conducted in a ProFlex™ 96-well PCR System, and quantitative PCR was performed using a QuantStudio 5 Real-Time PCR System (Applied Biosciences™, Thermo Fisher Scientific, Waltham, MA, USA). β-Actin levels were used to normalize gene expression across samples, and fold changes were calculated using the 2^−ΔΔCt^ method.

### 2.16. Statistical Analyses

All data are presented as mean ± standard deviation (SD) for all mice (*n* = 4 per group) or at least three independent experiments (*n* ≥ 3). Statistical analyses were performed using GraphPad Prism 11 (GraphPad Software Inc., San Diego, CA, USA). Specific tests included Welsh’s unpaired *t*-test, one-way ANOVA with Dunnett’s multiple comparison test, or two-way ANOVA with Šidák’s multiple comparison test, as detailed in the relevant figure legends. Correlation was calculated using Pearson coefficient. Statistical significance was set at α = 0.05 with a 95% confidence interval and denoted as follows: * *p* < 0.05, ** *p* < 0.01, *** *p* < 0.001, and **** *p* < 0.0001.

## 3. Results

### 3.1. Body Weight Gain and Metabolic Health After High-Fat Diet Exposure in C57BL/6 Mice

**Figure 1:**
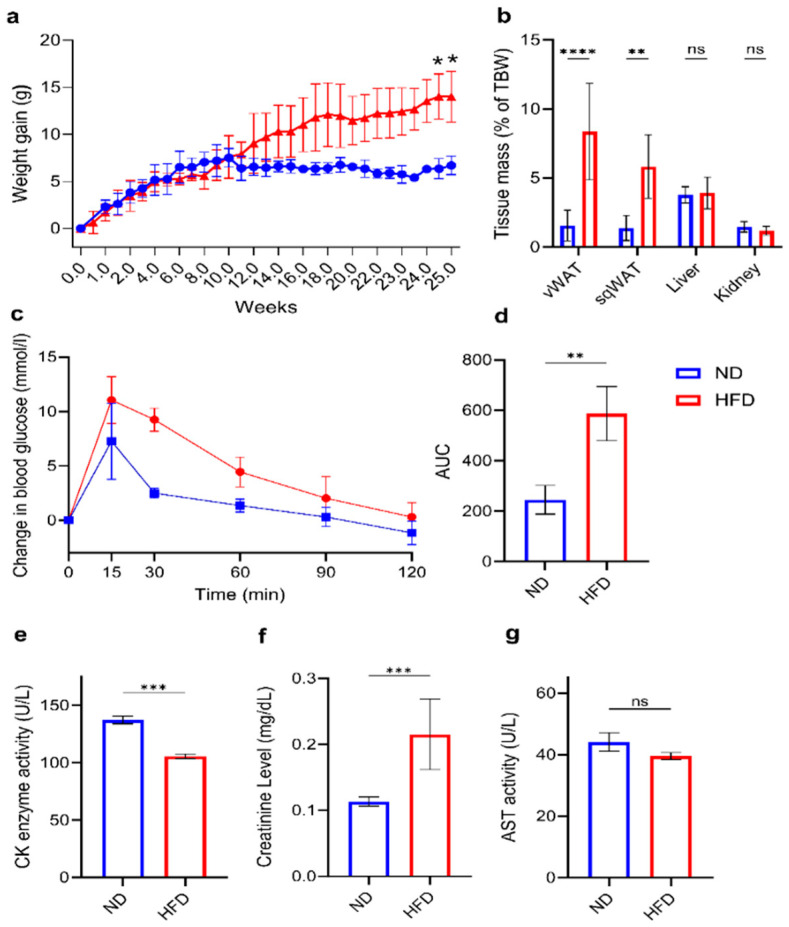
High-fat diet induces obesity and metabolic impairment in C57BL/6 mice. Male C57BL/6 mice were fed a normal diet (ND; blue) or a high-fat diet (HFD; red) for 25 weeks. (**a**) Mean weight gain ± SD. (**b**) Tissue masses expressed as a percentage of total body weight (TBW) for visceral white adipose tissue (vWAT), subcutaneous WAT (sqWAT), liver, and kidney (mean ± SD). (**c**) Intraperitoneal glucose tolerance test (IPGTT) showing blood glucose levels over time (mean ± SD) and (**d**) corresponding area under the curve (AUC). Serum levels of (**e**) creatine kinase (CK) activity, (**f**) creatinine, and (**g**) aspartate aminotransferase (AST) activity (mean ± SD). All data are from *n* = 4 biologically independent mice per group. Data were analyzed by unpaired *t*-test (**a**,**c**–**g**) or two-way ANOVA with Šidák’s multiple comparison test (**b**). ns, not significant, * *p* < 0.05, ** *p* < 0.01, *** *p* < 0.001, and **** *p* < 0.0001.

To establish the DIO model, male C57BL/6 mice were fed an HFD comprising standard chow supplemented with a high-fat paste for 25 weeks. HFD-fed mice consumed an average of 1.1 g/day/mouse of the supplement and gained significantly more weight than ND controls (13.2 g vs. 6.7 g; *p* < 0.05; Figure 1a). This was accompanied by a significant increase in adipose tissue mass, with 6.8% more vWAT (*p* < 0.0001) and 4.4% more sqWAT (*p* < 0.01) relative to the total body weight (Figure 1b). The HFD group also exhibited systemic metabolic dysfunction. They displayed impaired glucose tolerance compared to ND-fed mice (*p* < 0.01; Figure 1c,d). Serum analyses revealed significantly reduced CK activity (*p* < 0.001; Figure 1e), suggesting obesity-associated muscle weakness [31,32]. Despite remaining within the normative range [33], serum creatinine levels were significantly elevated in HFD-fed mice (*p* < 0.001; Figure 1f), which may indicate early renal impairment. Serum AST activity, a marker of liver health, was 10.4% lower in the HFD-fed group; however, this difference was not statistically significant when compared to the ND-fed group (*p* = 0.055). Overall, HFD-fed mice demonstrated significant weight gain and reduced metabolic health, as is commonly seen in chronic models of obesity [16].

### 3.2. APP and APP-βCTF Levels Are Elevated in Adipose and Kidney Tissues of HFD-Fed Mice

**Figure 2:**
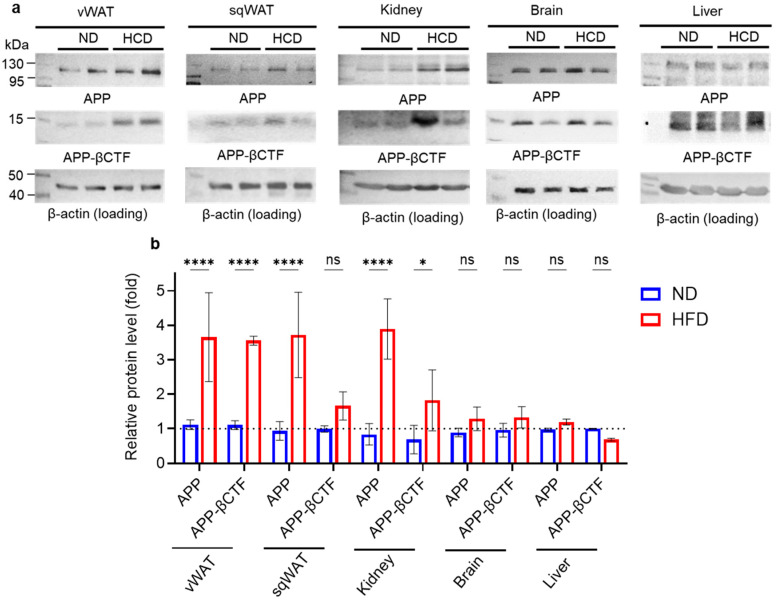
Amyloid precursor protein (APP) and APP-βC-terminal fragment (APP-βCTF) level after high-fat diet (HFD). (**a**) Representative Western blots of APP and APP-βCTF in visceral white adipose tissue (vWAT), subcutaneous white adipose tissue (sqWAT), kidney, brain, and liver from mice fed a normal diet (ND, blue) or HFD (red). (**b**) Quantification of APP (full-length) and APP-βCTF levels normalized to β-actin (mean ± SD; *n* = 4 mice per group). Data were analyzed by two-way ANOVA with Šidák’s multiple comparison test. Dotted line indicates control protein level of 1-fold. ns, not significant, * *p* < 0.05, and **** *p* < 0.0001.

We next assessed the impact of an HFD on APP and its proteolytic fragments across multiple tissues. Quantitative Western blot analysis revealed that HFD triggered tissue-specific APP overexpression. Significant increases in APP levels were observed in both vWAT and sqWAT (2-fold; *p* < 0.0001) and in kidney tissue (2.9-fold; *p* < 0.0001) (Figure 2). In contrast, APP levels in the brain and liver remain unchanged, indicating that this upregulation is not a systematic response [5]. We subsequently investigated whether this APP overexpression led to an accumulation of its CTFs. A prominent 14 kDa band, consistent with the size of APP-βCTF [23], was detected. The levels of APP-βCTF were significantly elevated in the vWAT (2.5-fold; *p* < 0.0001) and kidney (0.8-fold; *p* < 0.05) from HFD-fed mice (Figure 2). A similar, though non-significant, increase was observed in sqWAT (0.7-fold; *p* = 0.57). These findings demonstrate that HFD promotes the accumulation of both full-length APP and APP-βCTF in a tissue-specific manner and that βCTF production is regulated by factors beyond just APP abundance.

### 3.3. HFD Induces Mitochondrial Accumulation of APP and Its βCTF Fragment and Compromises Electron Transport Chain Activity

**Figure 3:**
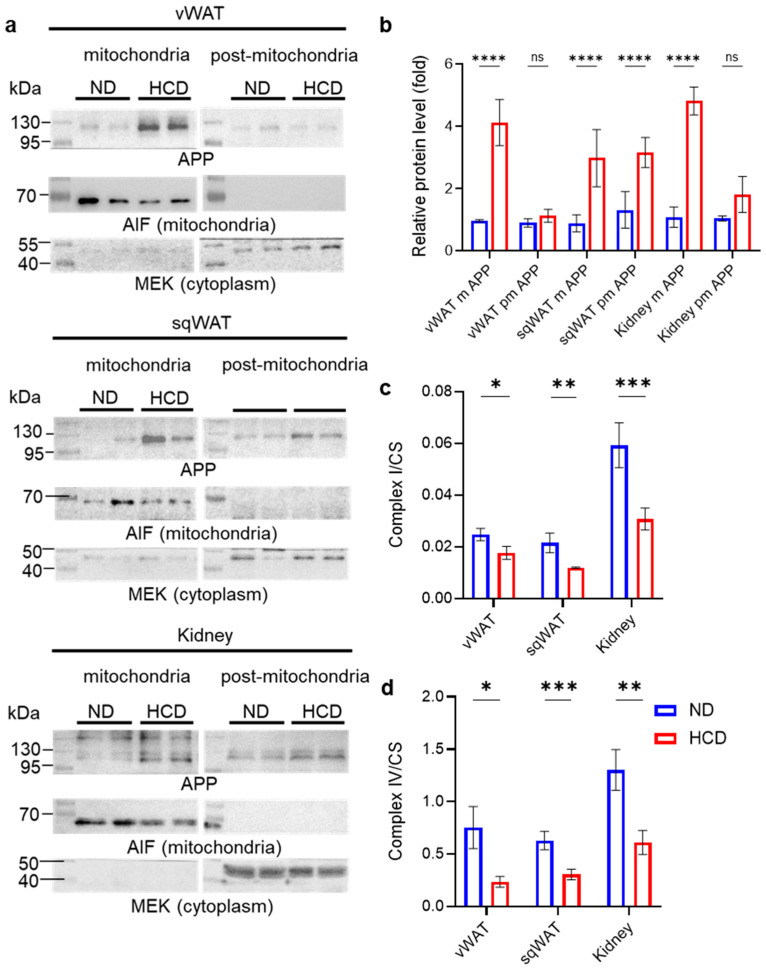
High-fat diet (HFD) induces mitochondrial APP accumulation and impairs complex I and IV activity in vivo. (**a**) Representative Western blots of APP in mitochondrial (m) and post-mitochondrial (pm) fractions from visceral white adipose tissue (vWAT), subcutaneous white adipose tissue (sqWAT), and kidney of mice fed a normal diet (ND) or HFD. (**b**) Quantification of APP levels in mitochondrial and post-mitochondrial fractions normalized to organelle-specific markers (ND, blue; HFD, red). (**c**) Complex I/citrate synthase (CS) activity ratios. (**d**) Complex IV/CS activity ratios. All quantitative data (**b**–**d**) are presented as mean ± SD (*n* = 4 mice per group) and were analyzed by two-way ANOVA with Šidák’s multiple comparison test. * *p* < 0.05, ** *p* < 0.01, *** *p* < 0.001, and **** *p* < 0.0001; ns, not significant.

Building on previous observations in neuronal and adipocyte mitochondria [5,8], we investigated whether APP accumulates in the mitochondria of tissues affected by an HFD. We found a pronounced (>3-fold) enrichment of APP in the mitochondrial fractions of the vWAT, sqWAT, and kidney from obese mice (Figure 3a,b). In the vWAT and kidney, this overexpressed APP was preferentially localized to the mitochondria, with no significant increase in the post-mitochondrial fraction (containing cytoplasm, plasma membrane, Golgi, and endosomes) (Figure 3a,b). Although the APP-βCTF was less abundant than full-length APP, its mitochondrial levels were significantly elevated in the vWAT (3.9-fold; *p* < 0.001) and kidney (2.6-fold; *p* < 0.05) from HFD-fed mice, with no change in the corresponding post-mitochondrial fractions (Figure S1).

This mitochondrial accumulation of APP coincided with functional defects. The specific activity of complex I relative to mitochondrial abundance, measured in isolated mitochondria, was significantly reduced in vWAT, sqWAT, and kidney tissue from HFD-fed mice (Figure 3c). Analysis of total tissue lysates revealed that complex IV impairment was specific to tissues exhibiting APP overexpression (vWAT, sqWAT, and kidney), with no significant deficit observed in the brain or liver where APP levels were unchanged (Figure 3d and Figure S2a). Moreover, a positive correlation between the decrease in complex I (r = 0.6712, *p* < 0.05) and IV (r = 0.8946, *p* < 0.0001) activities and the level of mitochondrial APP observed in HFD-exposed vWAT, sqWAT, and kidney tissues was also apparent (Figure S3). Intriguingly, citrate synthase activity increased in the vWAT after an HFD (Figure S2b), suggesting a potential compensatory increase in mitochondrial mass in response to functional impairment [34]. Thus, HFD exposure resulted in the co-occurrence of elevated mitochondrial APP and compromised mitochondrial function.

### 3.4. APP Overexpression Is Sufficient to Disrupt Mitochondrial Function In Vitro

**Figure 4:**
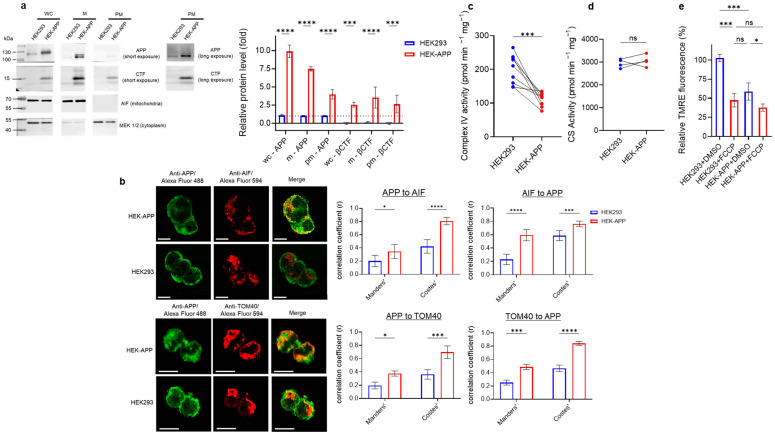
APP overexpression drives mitochondrial mislocalization and dysfunction in vitro. (**a**) Representative Western blots and quantification of APP and APP-βCTF in whole-cell (WC), mitochondrial (m), and post-mitochondrial (pm) fractions from the HEK293 and HEK-APP cell lines. Protein levels were normalized as described in the Methods (mean ± SD; *n* ≥ 3). Dotted line indicates control protein level of 1-fold. (**b**) Confocal microscopy images showing APP (green) and mitochondrial markers AIF or TOM40 (red). Scale bar: 10 µm. Manders’ and Costes’ correlation coefficients are shown (mean ± SD). (**c**) Complex IV and (**d**) citrate synthase (CS) activity. (**e**) Mitochondrial membrane potential (ΔΨm) measured by TMRE fluorescence; FCCP as depolarization control. Data were analyzed by two-way ANOVA with Šidák’s multiple comparison test (**a**), one-way ANOVA with Dunnett’s multiple comparison test (**b**,**e**), or an unpaired *t*-test (**c**,**d**). All data are plotted as mean ± SD. For all panels, * *p* < 0.05, *** *p* < 0.001, and **** *p* < 0.0001; ns, not significant.

**Figure 5:**
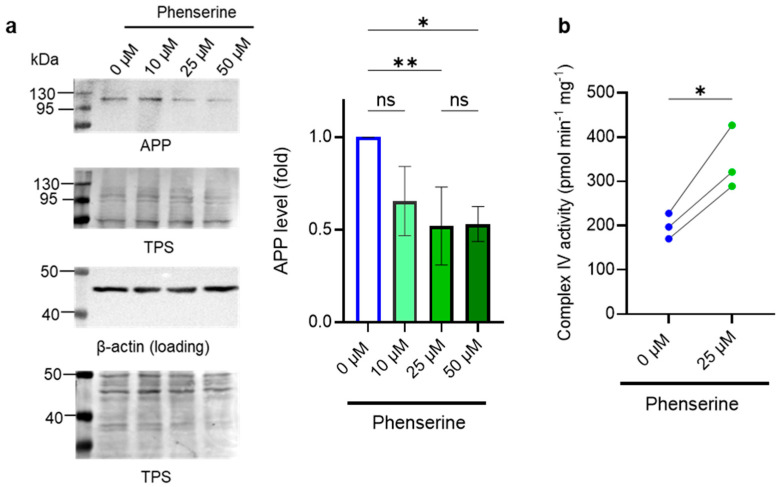
Pharmacological reduction in APP enhances mitochondrial complex IV activity. The HEK293 cell line was treated with phenserine (0–50 µM) for 24 h. (**a**) Representative Western blots with total protein stain (TPS) and corresponding quantification of APP levels normalized to β-actin. (**b**) Complex IV activity following APP reduction. Data are presented as mean ± SD (*n* ≥ 3) and were analyzed by one-way ANOVA with Dunnett’s multiple comparison test (**a**) or unpaired *t*-test (**b**). * *p* < 0.05, ** *p* < 0.01; ns, non-significant.

To establish a causal relationship between APP levels and mitochondrial function, we compared an HEK-APP overexpressing cell line [21] with the parental HEK293 cell line. APP levels were 8.9-fold higher in the HEK-APP cell line (Figure 4a). Recapitulating our in vivo findings, the excess APP predominantly accumulated in the mitochondrial fraction, accounting for 71.9% of the total cellular increase, with a smaller increase (32.6%) in the post-mitochondrial fraction (Figure 4a). APP-βCTF, which was undetectable in control cells, appeared in all fractions of the HEK-APP line (Figure 4a). Confocal microscopy confirmed the mitochondrial mislocalization, demonstrating the significantly increased colocalization of APP/APP-βCTF with the mitochondrial markers AIF and TOM40 (Figure 4b). The aberrant localization compromised mitochondrial integrity. The HEK-APP cell line exhibited significantly reduced complex IV activity and mitochondrial membrane potential (ΔΨm), with only minor changes in mitochondrial abundance (Figure 4c–e). To confirm the specificity of this effect, we reduced APP levels in the parental HEK293 line using phenserine. The HEK293 cell line—known to have high-levels of endogenous APP—in comparison, expresses little to none of the other described phenserine targets: acetylcholinesterase, α-synuclein, and huntingtin [35,36,37,38,39]. A ~50% reduction in APP at 25 µM [40] significantly increased complex IV activity (*p* < 0.05; Figure 5). Collectively, these data demonstrate that APP overexpression is sufficient to drive mitochondrial localization and the subsequent mitochondrial dysfunction.

### 3.5. HFD Alters the Cytokine Profile of Adipose and Kidney Tissue

**Figure 6:**
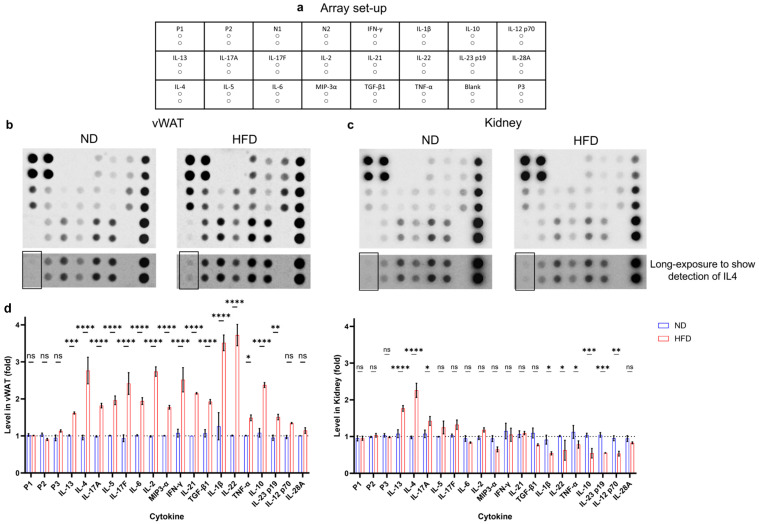
High-fat diet induces distinct cytokine profiles in white adipose and kidney tissue. (**a**) Layout of the cytokine array membrane, showing the position of each cytokine in paired dots, with positive (P1–P3) and negative (N1–N2) controls. Representative array blots for (**b**) visceral white adipose tissue (vWAT) and (**c**) kidney from mice fed a normal diet (ND; blue) or high-fat diet (HFD; red). Box indicates IL-4 detection. (**d**) Quantification of cytokine levels normalized as described in the Methods (mean ± SD). Dotted line indicates control protein level of 1-fold. Data were analyzed by two-way ANOVA with Šidák’s multiple comparison test. * *p* < 0.05, ** *p* < 0.01, *** *p* < 0.001, and **** *p* < 0.0001; ns, not significant.

To profile the systematic inflammatory response, we performed a cytokine array (Figure 6) on the vWAT and kidney from ND- and HFD-fed mice, quantifying changes across 18 cytokines. Consistent with the established pattern of diet-induced inflammation, HFD feeding provoked a robust inflammatory response in vWAT, significantly altering 16 of the 18 cytokines profiled (Figure 6b,d). The most pronounced elevations were observed for IL-1β and IL-22 (>2-fold; *p* < 0.0001). Furthermore, we detected a significant upregulation of IL-4 (1.8-fold) and its functional homolog IL-13 (*p* < 0.0001 and 0.001, respectively), underscoring a potential role for Th2-associated cytokines in the adipose tissue response to an HFD. In contrast, the kidney exhibited a more restrained and unique cytokine profile. Only three cytokines were significantly elevated, with IL-4 showing the most substantial increase (1.3-fold), followed by IL-13 and IL-17A (Figure 6c,d). Notably, six cytokines were significantly suppressed in the kidney, five of which—IL-1β, IL-22, TNFα, IL-10 and IL 23p19—were concurrently elevated in the vWAT. These findings demonstrate that an HFD disrupts cytokine homeostasis in both adipose and kidney tissues. The markedly different cytokine profiles between these tissues reveal a delayed or tissue-specific response to this metabolic stress.

### 3.6. Cytokines That Are Upregulated After HFD Exposure Increase APP mRNA Level

**Figure 7:**
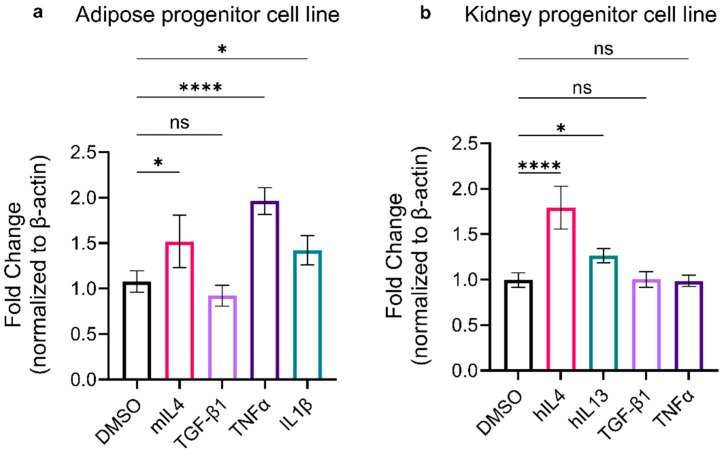
Pro-inflammatory cytokines upregulate *APP* mRNA expression in vitro. (**a**) 3T3-L1 preadipocytes were treated with mIL-4, TGF-β1, TNF-α, or IL1-β (5 ng/mL). (**b**) HEK293 cells were treated with hIL-4, hIL-13, TGF-β1, or TNFα (5 ng/mL). Cells were exposed to cytokines or DMSO vehicle for 24 h, and mRNA levels were quantified by qPCR. Data were normalized to β-actin and are presented as fold change relative to the DMSO control (mean ± SD; *n* ≥ 3). Statistical significance was determined by one-way ANOVA with Dunnett’s multiple comparison test. * *p* < 0.05, **** *p* < 0.0001; ns, not significant.

Given the established correlation between inflammation and APP overexpression [1,2,3,4,5], we investigated whether cytokines upregulated by HFD could directly stimulate *APP* transcription. In 3T3-L1 preadipocytes, treatment with mIL-4, TNFα, or IL-1β significantly increased *APP* mRNA levels by 42–77% (*p* < 0.05, *p* < 0.0001, and *p* < 0.05, respectively; Figure 7a). Similarly, in the HEK293 cell line, exposure to hIL-4 or hIL-13 elevated *APP* mRNA by 22–80% (*p* < 0.0001 and *p* < 0.05, respectively; Figure 7b). TGF-β1 had no effect in either cell line, and IL-1β could not be tested in HEK293 cells due to a lack of the required IL-1R1 [41]. These results identify TNF-α, IL-1β, and the IL-4/IL-13 pair as direct regulators of *APP* transcription. Together with our cytokine array data, these findings provide a mechanistic link between HFD-induced inflammation and the tissue-specific APP upregulation observed in adipose and kidney tissue.

## 4. Discussion

Our study delineates a tissue-specific pattern of APP dysregulation in a DIO model. We confirm that a prolonged HFD induces significant APP overexpression in the vWAT and sqWAT and critically identify the kidney as a new and responsive peripheral site. This renal-associated APP accumulation is particularly interesting given the concurrent elevation of serum creatinine in our model, an early marker of renal impairment. In contrast, APP levels in whole brain and liver were unchanged, underscoring a selective response to this metabolic stress. While APP upregulation in WAT is a consistent outcome of an HFD [5,6,7], its regulation elsewhere is less defined and appears context-dependent. For instance, although an acute HFD does not alter total brain *APP* mRNA [5]—a finding we extend to protein levels after chronic feeding—hippocampal APP can be elevated by prolonged HFD exposure [7], indicating regional neuronal specificity. The hippocampus—rich in microglia and astrocytes—is particularly susceptible to HFD-induced neuroinflammation, which may account for this specificity [42,43,44]. Similarly, despite HFD-induced hepatic dysfunction [3,16,45,46] and the induction of hepatic APP by chronic alcohol intake [3,16,45,46], neither an acute [5] nor chronic (this study) HFD upregulates the liver APP. The differential hepatic overexpression of APP may reflect the nature of the underlying stimulus, as alcohol and HFD induce distinct inflammatory responses. Chronic alcohol exposure activates Kupffer cells via the liver–gut axis, promoting a neutrophil-rich response [47], whereas HFD induces lipotoxic hepatocyte stress and a macrophage-dominated profile [48]. This suggests that HFD-associated inflammation in the liver may be less effective in driving APP overexpression. Our identification of renal APP overexpression expands the known repertoire of tissues susceptible to APP dysregulation in the HFD model. To further characterize this dysregulation, the pancreas is a key organ for future studies due to its sensitivity to HFD-induced inflammation and its baseline APP expression [35,36,49].

In these susceptible tissues, APP overexpression coincided with impaired mitochondrial electron chain enzymes. Our initial analysis of whole-tissue lysates revealed that APP was significantly elevated in vWAT, sqWAT, and kidney from obese mice. βCTF was significantly elevated in the vWAT and kidney only, indicating that its accumulation in vivo is gated by specific factors like secretase activity rather than being a passive stoichiometric byproduct of APP overexpression. Both full-length APP and the βCTF pool accumulated in the mitochondria—a phenomenon we extend from neuronal systems [10,11] to peripheral tissues and cells. This mislocalization may have functional consequences: mitochondrial APP enrichment correlated with reduced complex I and IV activities in adipose and kidney tissues in vivo. Our in vitro studies further confirmed that APP overexpression drove mitochondrial accumulation and impaired complex IV activity, while pharmacological reduction in APP levels restored enzyme function. Given that βCTF itself is an established mitochondrial toxin [10], we suggest that HFD-triggered APP and βCTF accumulation within the mitochondria may jointly impair electron transport and contribute to the core mitochondrial pathology of obesity.

We further identified a plausible inflammatory trigger for this APP dysregulation. Cytokine profiling revealed that an HFD induced markedly distinct inflammatory landscapes in the WAT and kidney. While the WAT exhibited a broad inflammatory response, the kidney showed a more restrained profile, with significant upregulation of only three cytokines. Notably, IL-4, IL-13, and IL-17A were also elevated in the WAT. Functionally, we demonstrated that the IL-4/IL-13 pair directly upregulated *APP* mRNA in both preadipocyte and kidney progenitor cell lines in vitro. This is significant in the context of obesity, where IL-4 is elevated in the WAT early after HFD initiation [50], and its signaling exacerbates weight gain and metabolic comorbidities [51,52]. While other cytokines such as TNF-α and IL-1β also increased the APP in preadipocytes [53,54], TGF-β1 had no effect, highlighting a cell-type-specific regulatory network. Collectively, our work corroborates the model in which a cytokine-driven inflammatory response may be a key driver of APP overexpression in metabolic tissues after HFD exposure [5,7,54]. Our findings implicate the IL-4/IL-13 cytokine pair as novel contributors to this phenomenon. Although the activation of immune cells, via the IL-4 type I receptor, by IL-4 is generally classified as an anti-inflammatory response for the promotion of cell proliferation and survival, the role of IL-4 in non-hematopoietic cells is pro-inflammatory [55,56]. This, via the activation of the IL-4 type II receptor by either IL-4 or IL-13, has a prominent role in allergic inflammation [55]. The precise mechanisms by which IL-4 and IL-13 may regulate APP expression have yet to be fully elucidated. However, given that IL-4 and IL-13 can activate the Akt/NF-κB and Ras/MAPK pathways—both of which are known to regulate *APP* transcription—these signaling cascades represent promising avenues for future investigation [53,56].

Collectively, our findings demonstrate that APP is overexpressed in response to DIO in select peripheral tissues. Specifically, an HFD induces APP overexpression in adipose tissue and, notably, in the kidney, where it may correlate with initial renal impairment. This APP dysregulation may drive mitochondrial dysfunction, as APP and its toxic βCTF fragment accumulate within the mitochondria, which coincides with impaired electron transport chain enzymes. Furthermore, we establish the potential cytokine drivers of *APP* transcription, linking diet-induced inflammation to APP overexpression.

## Data Availability

All data generated or analyzed during this study are included in this published article [and its Supplementary Information Files] or are available on the Zenodo repository: https://doi.org/10.5281/zenodo.19494547.

## Supplementary Materials

The following supporting information can be downloaded at: https://www.mdpi.com/article/10.3390/cells15111033/s1, Table S1: Nutritional information for the high calorie supplement; Figure S1: APP-βCTF accumulates in the mitochondrial fraction of visceral white adipose and kidney tissue after high-fat diet (HFD); Figure S2: Complex IV and citrate synthase (CS) activity in a panel of tissues after high-fat diet (HFD) exposure; Figure S3:Correlation between mitochondrial APP level and the decrease in complex I/CS and IV/CS activity after high-fat diet exposure; Data S1. Full-length Western blots.

## Abbreviations

The following abbreviations are used in this manuscript:

AD	Alzheimer’s disease
APP	amyloid precursor protein
AUC	area under the curve
β/CTF	β/C-terminal fragment
CK	creatine kinase
CS	citrate synthase
AST	aspartate aminotransferase
DIO	diet-induced obesity
FCCP	carbonyl cyanide 4-(trifluoromethoxy) phenylhydrazone
HFD	high fat diet
IPGTT	intraperitoneal glucose tolerance test
ND	normal diet
PBS(-T)	phosphate buffered saline (with tween 20)
PIC	protease inhibitor cocktail
RT	room temperature
SD	standard deviation
sqWAT	subcutaneous WAT
TMRE	tetramethyl rhodamine ethyl ester perchlorate
vWAT	visceral WAT
WAT	white adipose tissue
