# Supplementary material for: Diet-Induced Amyloid Precursor Protein Dysregulation in Kidney and Adipose Tissue Mediates Mitochondrial Dysfunction
Source: Cells. 2026 Jun 4;15(11):1033. doi: 10.3390/cells15111033 (PMC13256868; doi:10.3390/cells15111033)

Supplementary data file

Title page

Title: Diet-induced amyloid precursor protein dysregulation in kidney and adipose tissue mediates mitochondrial dysfunction.

Author Affiliation: Alexandré Delport[a], Ebrahim Ally[b], Shantal Maharaj[a], Raymond Hewer[a].

[a]Discipline of Biochemistry, School of Agriculture and Science, University of KwaZulu-Natal, Pietermaritzburg, South Africa, 3201.

[b]Animal House, School of Agriculture and Science, University of KwaZulu-Natal, Pietermaritzburg, South Africa, 3201.

ORCID: Alexandré Delport: 0000-0003-3511-4369, Shantal Maharaj: 0000-0002-9380-0028, Raymond Hewer: 0000-0002-6025-8826.

Corresponding Author: Dr Alexandré Delport

Telephone number: +27 33 260 5150
Email: delporta1@ukzn.ac.za

Nutritional Information

Table S1. Nutritional information for the high calorie supplement

	High Calorie Supplement[a]
Protein	10.7%
Carbohydrates	13.0%
Sugar	8.75%
Fat	63.45%
Sodium	0.445%

[a]Mice in the high calorie diet group consumed, on average, 1.13 ± 0.4 g/day of the supplement.

APP-βCTF level in the mitochondrial and post-mitochondrial fractions of adipose and kidney tissue

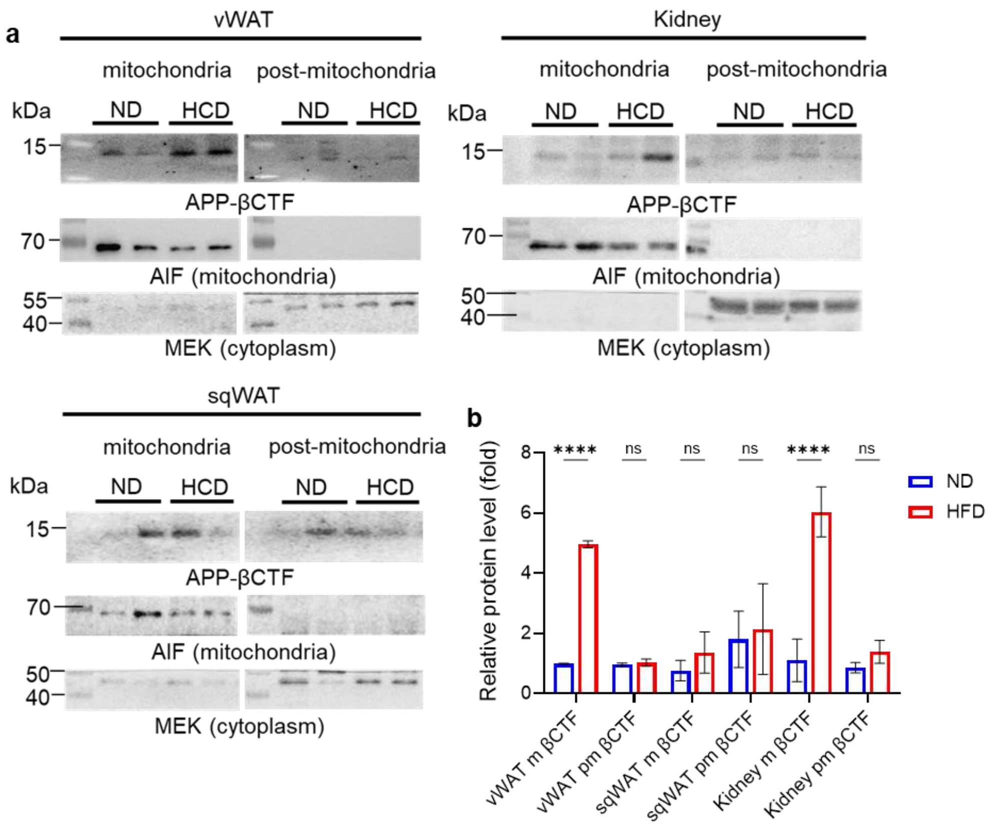

Figure S1. APP-βCTF accumulates in the mitochondrial fraction of visceral white adipose and kidney tissue after high-fat diet (HFD). (a) Representative western blots of subcellular fractions from visceral white adipose tissue (vWAT), subcutaneous white adipose tissue (sqWAT), and kidney of mice fed a normal diet (ND, blue) or a HFD (red), probed for βCTF. **(b)** Quantification of APP-βCTF levels in mitochondrial (m) and post-mitochondrial (pm) fractions, normalized as described in Methods (mean ± SD, n = 4 mice per group). Data were analyzed by two-way ANOVA with Šidák's multiple comparison test. ns, not significant and ****$p < 0.0001$.

Complex IV activity in brain and liver tissue and citrate synthase activity in vWAT, sqWAT and kidney after high fat diet exposure

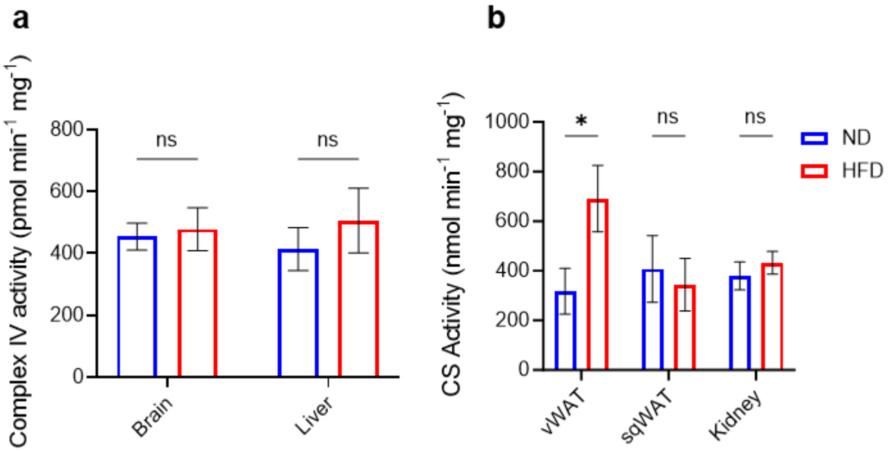

Figure S2. Complex IV and citrate synthase (CS) activity in a panel of tissues after high fat diet (HFD) exposure. (a) Complex IV activity from brain and liver tissue after normal diet (ND) or HFD exposure and **(b)** CS activity in visceral white adipose tissue (vWAT), subcutaneous white adipose tissue (sqWAT), and kidney tissue after ND or HFD exposure. All quantitative data are presented as mean ± SD (*n* = 4 mice per group) and were analysed by two-way ANOVA with Šidák's multiple comparisons test. ns, not significant and $^{**}p < 0.01$.

Correlation between mitochondrial APP level and the decrease in complex I/CS and IV/CS activity after HFD exposure.

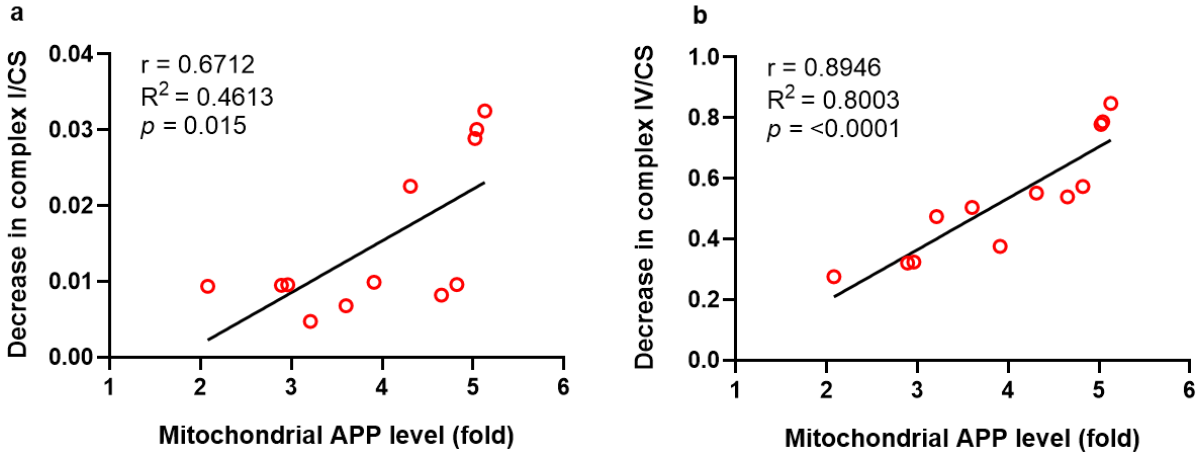

Figure S3. Correlation between mitochondrial APP level and the decrease in complex I/CS and IV/CS activity after high-fat diet exposure. The decrease in complex I/CS (a) or IV/CS (b) ratio was plotted against the corresponding mitochondrial APP level for visceral white adipose tissue (vWAT), subcutaneous white adipose tissue (sqWAT), and kidney tissue from each individual mouse in the HFD group. Pearson correlation coefficients were calculated in each case. Significance was taken as $p < 0.05$.

Data S1. Full-length western blots:

red blocks indicate portion of blots shown in final image

Full-length Western Blots – Figure S4

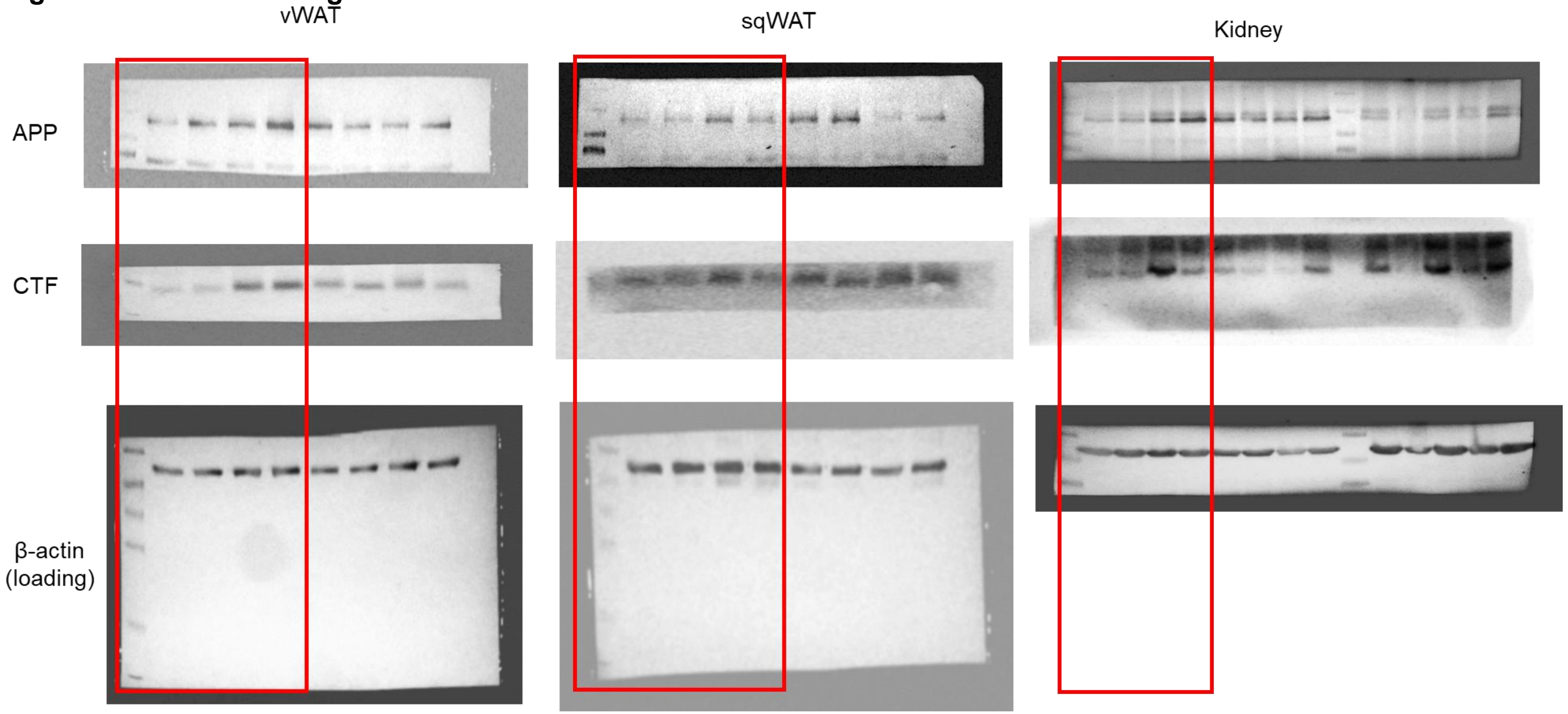

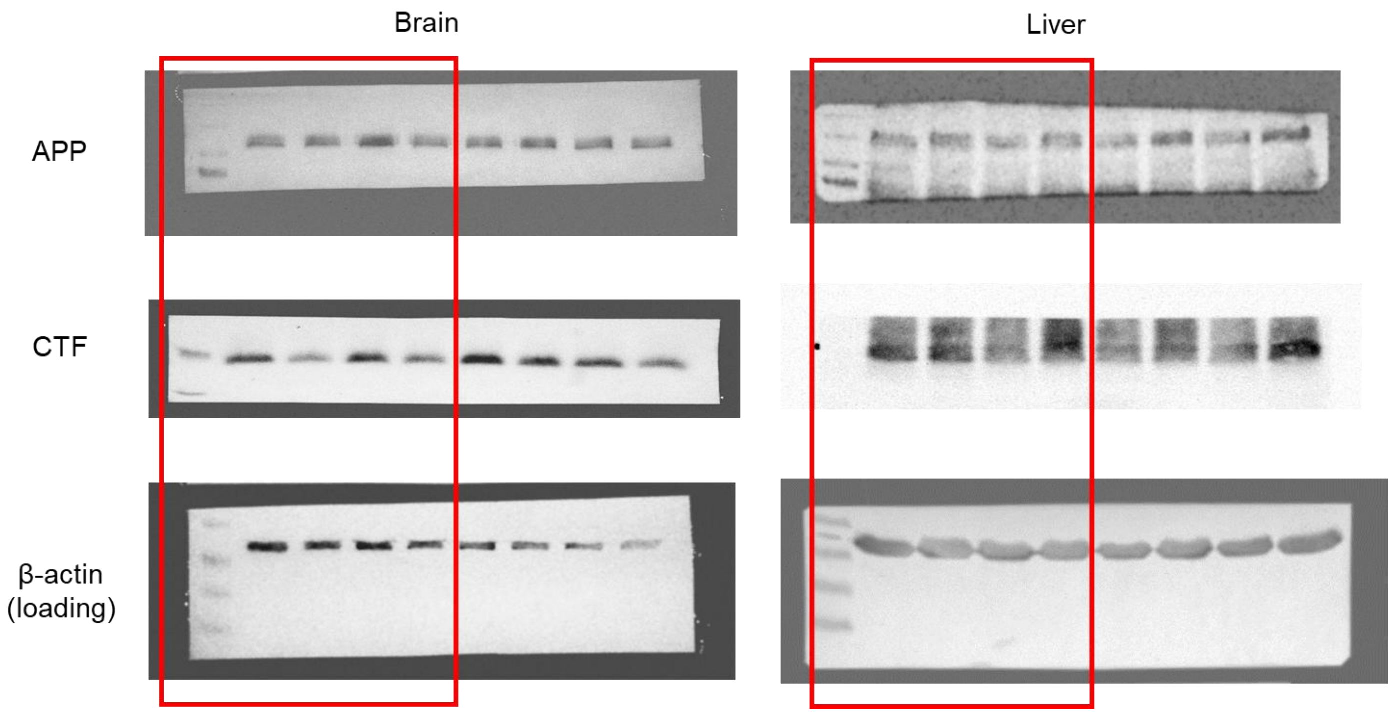
Brain
Liver
APP
CTF
β-actin
(loading)

Full-length Western Blots – Figure S5/S1

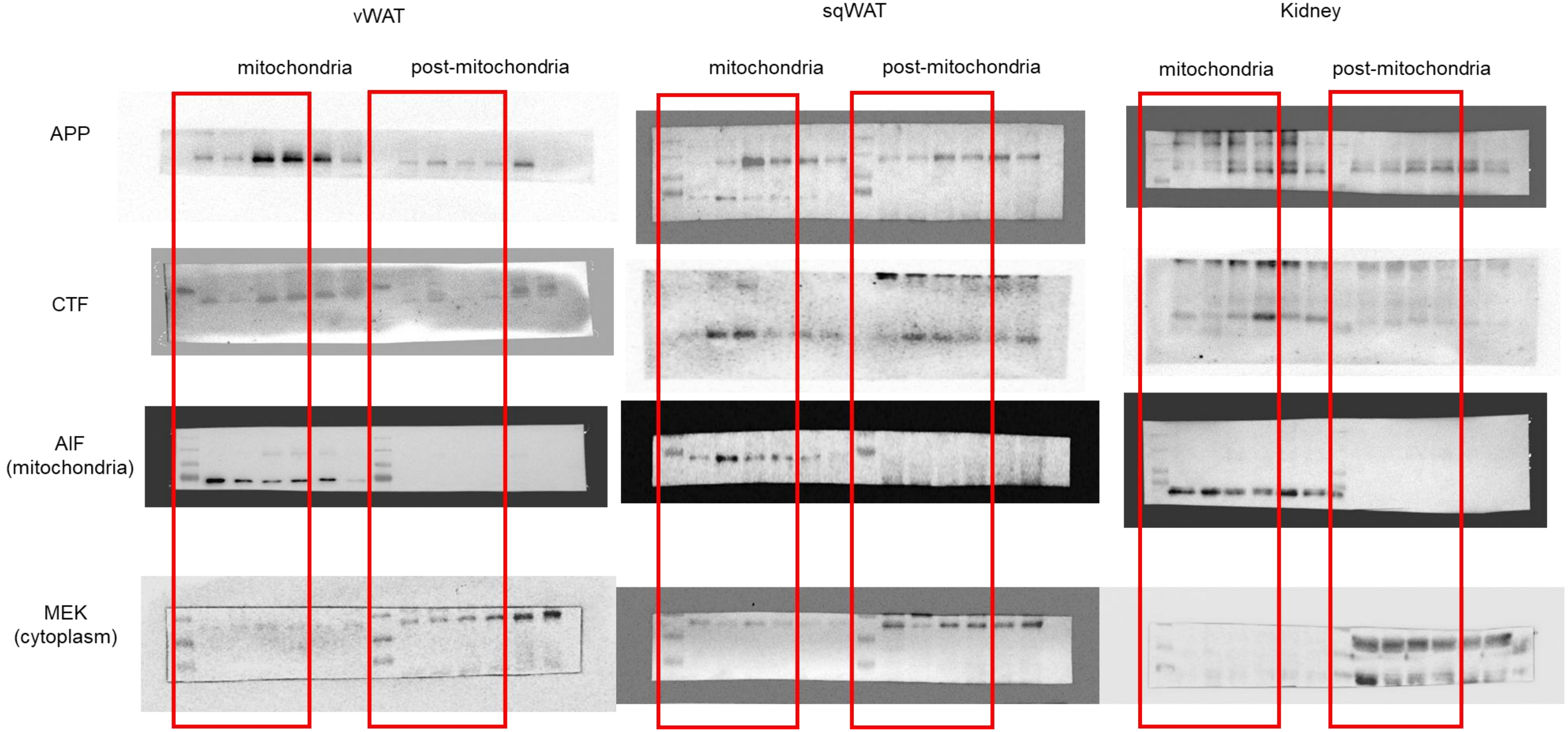

Full-length Western Blots – Figure S6

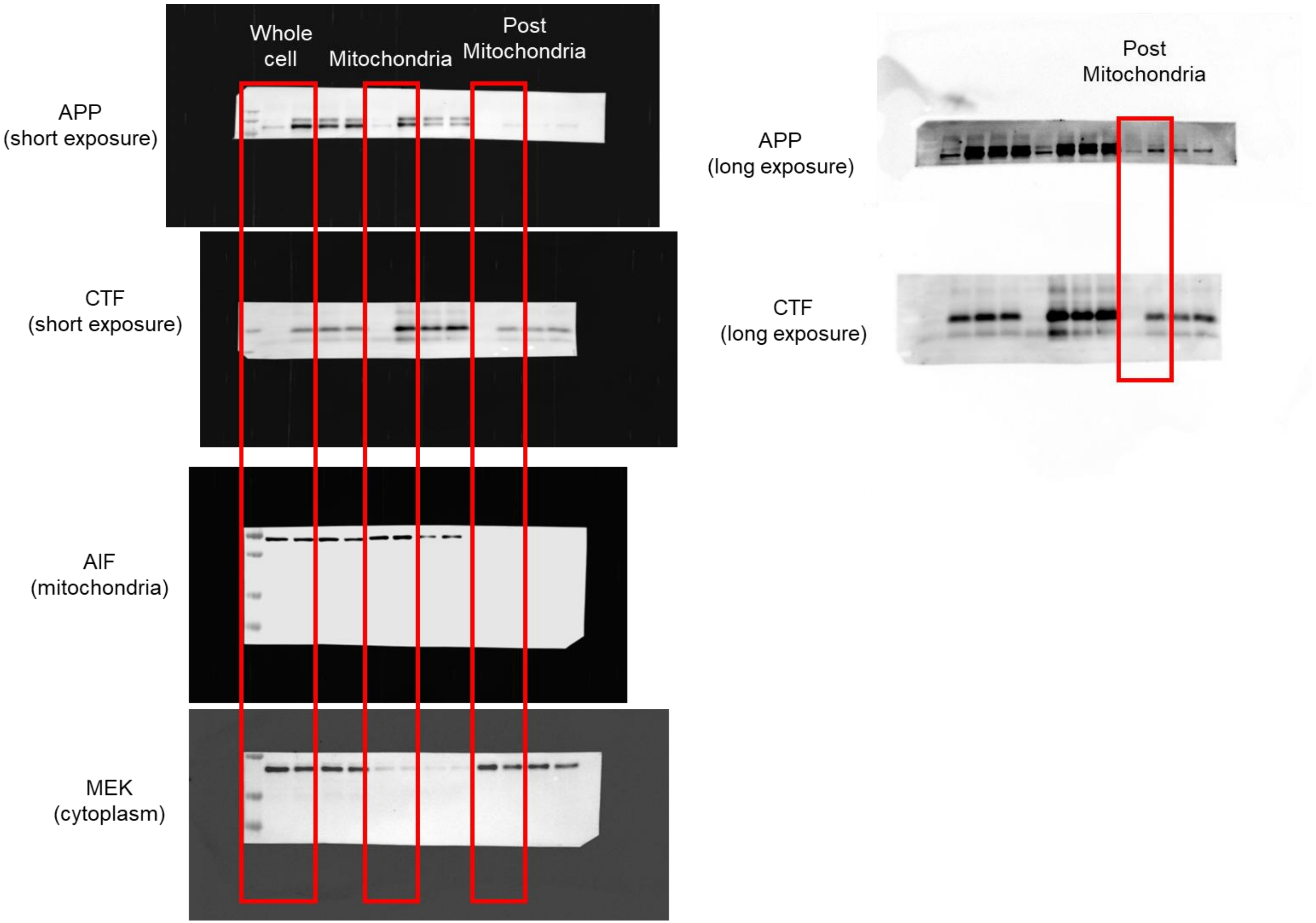

Full-length Western Blots – Figure S7

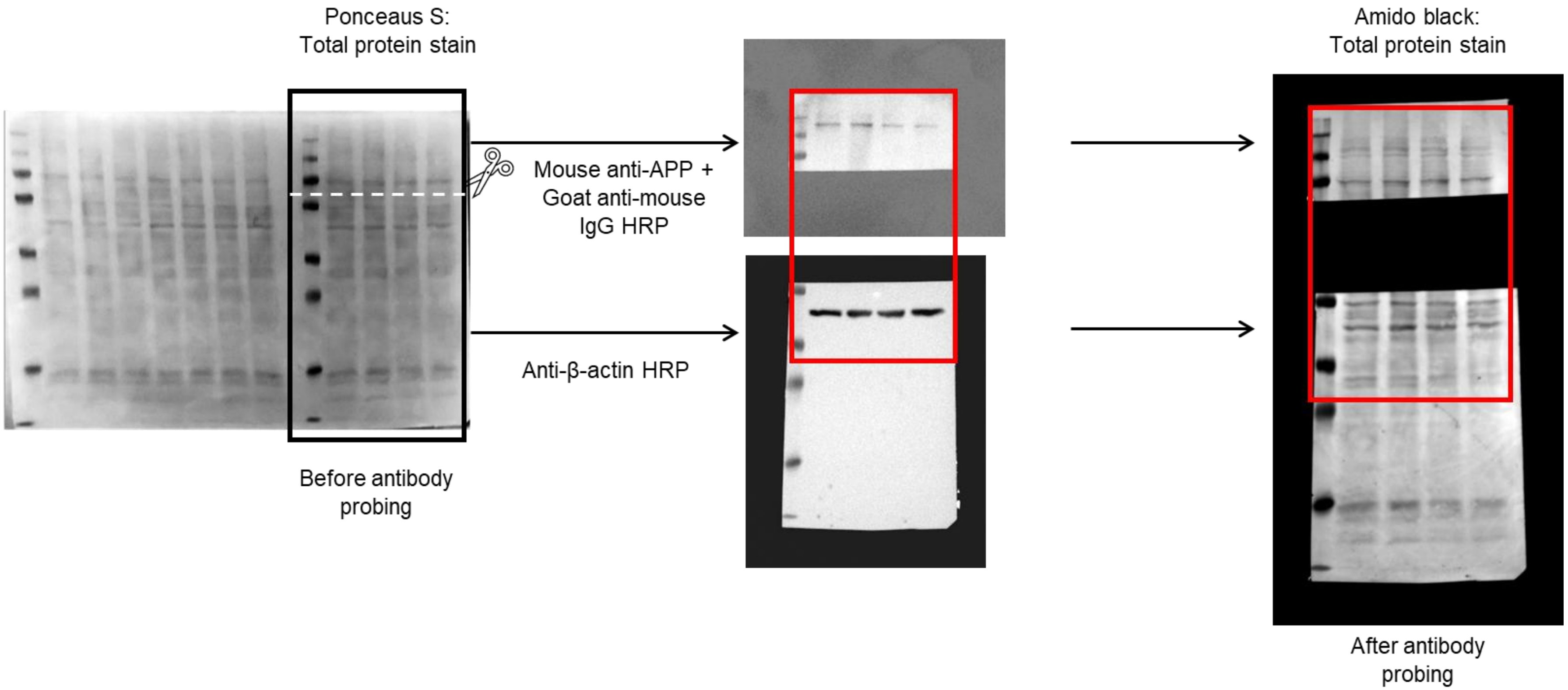

Supplement: Supplementary file 1 [file cells-15-01033-s001.zip › cells-4282041-supplementary.pdf]
